# Metabolomic Profiling and Machine Learning Models for Tumor Classification in Patients with Recurrent IDH-Wild-Type Glioblastoma: A Prospective Study

**DOI:** 10.3390/cancers16223856

**Published:** 2024-11-17

**Authors:** Rawad Hodeify, Nina Yu, Meenakshisundaram Balasubramaniam, Felipe Godinez, Yin Liu, Orwa Aboud

**Affiliations:** 1 Department of Biotechnology, School of Arts and Sciences, American University of Ras Al Khaimah, Ras Al Khaimah 72603, United Arab Emirates; 2 School of Medicine, University of California, Davis, Sacramento, CA 95817, USA; 3 Reynolds Institute on Aging, Department of Geriatrics, University of Arkansas for Medical Sciences, Little Rock, AR 72205, USA; 4 UC Davis Comprehensive Cancer Center, University of California, Davis, Sacramento, CA 95817, USA; 5 Department of Radiology, University of California, Davis, Sacramento, CA 95817, USA; 6 Department of Ophthalmology & Vision Science, University of California, Davis, Sacramento, CA 95817, USA; 7 Departments of Neurology and Neurological Surgery, University of California, Davis, Sacramento, CA 95817, USA

**Keywords:** glioblastoma, machine learning, metabolomics, recurrence

## Abstract

Background/Objectives: The recurrence of glioblastoma is an inevitable event in this disease’s course. In this study, we sought to identify the metabolomic signature in patients with recurrent glioblastomas undergoing surgery and radiation therapy. Methods: Blood samples collected prospectively from six patients with recurrent IDH-wildtype glioblastoma who underwent one surgery at diagnosis and a second surgery at relapse were analyzed using untargeted gas chromatography–time-of-flight mass spectrometry to measure metabolite abundance. The data analysis techniques included univariate analysis, correlation analysis, and a sample *t*-test. For predictive modeling, machine learning (ML) algorithms such as multinomial logistic regression, gradient boosting, and random forest were applied to predict the classification of samples in the correct treatment phase. Results: Comparing samples after the first surgery and after the relapse surgeries to the pre-operative samples showed a significant decrease in sorbitol and mannitol; there was a significant increase in urea, oxoproline, glucose, and alanine. After chemoradiation, two metabolites, erythritol and 6-deoxyglucitol, showed a decrease, with a cut-off of three and a significant reduction for 6-deoxyglucitol, while 2,4-difluorotoluene and 9-myristoleate showed an increase post radiation, with a fold-change cut-off of three. The gradient-boosting ML model achieved a high performance for the prediction of tumor conditions in patients with glioblastoma who had undergone relapse surgery. Conclusions: We developed an ML predictor for tumor phase based on the plasma metabolomic profile. Our study suggests the potential of combining metabolomics with ML as a new tool to stratify the risk of tumor progression in patients with glioblastoma.

## Simple Summary

Glioblastoma is a highly aggressive brain tumor, and its recurrence is common, leading to poor patient outcomes. This study aimed to understand the changes in the metabolic profile of patients with recurrent glioblastoma after undergoing surgery and radiation therapy. By analyzing blood samples from these patients, we identified specific metabolites that changed after tumor removal and radiation therapy. We then applied machine learning models to predict tumor progression. The gradient-boosting model demonstrated 92% accuracy for the prediction of tumor conditions in patients with glioblastoma who underwent relapse surgery. Our findings suggest that combining metabolomics with machine learning could offer a new way to predict and manage the progression of glioblastoma, potentially improving patient outcomes.

## 1. Introduction

IDH-wildtype glioblastomas are fatal central nervous system cancers [1]. The World Health Organization (WHO)’s 2021 classified IDH-wildtype glioblastomas as a grade 4 adult-type diffuse glioma that does not harbor mutations in IDH, but usually harbors mutations in the telomerase reverse transcriptase (*TERT*) promoter, leading to the gain of chromosomes 7 and the loss of chromosome 10, and in the epidermal growth factor receptor (*EGFR*) [2]. The WHO’s departure from histology as the primary classification determinant for glioblastomas and the integration of molecular markers signal the importance of molecular markers in supporting diagnosis, prognosis, and treatment.

Despite our growing knowledge about glioblastomas, there remains a proclivity for these neoplasms to recur and be difficult pathologies to manage. IDH-wildtype glioblastomas reappear in more than 80% of cases, making an already devastating diagnosis even more challenging to navigate for patients and their families [3]. Accumulated evidence has shown that intra-tumor heterogeneity contributes to the tumor’s aggressive behavior, recurrence, and poor prognosis [4,5,6]. An interesting study by He et al. [7] identified ubiquitous metabolites in necrotic and viable regions of glioblastoma tumors. Many of the identified metabolites belong to the tryptophan metabolic pathway. GB cells utilize multiple sources of energy, primarily through aerobic glycolysis and the tricarboxylic acid (TCA) cycle. Additionally, biosynthetic pathways such as the pentose phosphate pathway and serine and lipid biosynthesis are essential [8], as they provide necessary cofactors like nicotinamide adenine dinucleotide (NAD), which supports various metabolic processes. High levels of NAD have been associated with the resistance of glioma stem cells (GSCs) to radiation [9,10]. Recent findings indicate that the transfer of nicotinamide phosphoribosyltransferase (NAMPT) through microvesicles from radio-resistant glioma stem cells can enhance intracellular NAD+ levels in recipient cells, thereby promoting their resistance to radiation therapy [11]. Lucena-Cacace et al. demonstrated that elevated NAD+ levels are associated with increased nicotinamide phosphoribosyltransferase (NAMPT) activity, which correlates with tumor progression and a poor prognosis [12]. Metabolomics serve as an excellent field for better understanding the unique processes behind aberrant pathways involved in IDH-wildtype glioblastoma growth and progression. Machine learning can augment and optimize samples of metabolites to provide actionable data. Our previous work has shown that machine learning may advance our understanding of the metabolism of IDH-wildtype glioblastomas [13]. However, our previous analysis was based on the metabolomic profile of patients at the time of initial diagnosis and not at recurrence. Glioblastoma recurrence has been associated with a poor prognosis [14]. A prospective study by Sastry et al. on a cohort of 368 patients reported a trend for improvement in the survival rate by tumor resection, although the difference was not statistically significant. The median post-progression survival time for patients who underwent resection increased from 7.0 months to 12.8 months [15]. An interesting study by Mireles et al. in Norway demonstrated a significant increase in the survival rate of patients with glioblastoma who underwent contrast-enhancing tumor resection [16]. Although the effect of resection at glioblastoma recurrence on survival remains equivocal depending on the population and evolving medical imaging, the benefit of the correct and early detection of recurrence and reresection on patient outcome and quality of life remain important [17].

In this prospective study, we sought to identify metabolite changes that may be associated with recurrent IDH-wildtype glioblastomas in patients who underwent standard-of-care therapy including surgery and chemoradiation. We hypothesize that several serum metabolites are different in patients with recurrent glioblastoma before the first and relapse surgeries and after surgery and before and after chemoradiation. We further leveraged ML tools on whole-metabolomic data to develop an ML model that is able to stratify patients with glioblastoma recurrence with a high accuracy. We propose that such an approach could provide valuable information.

## 2. Materials and Methods

### 2.1. Patients

Six patients with recurrent IDH-wildtype glioblastoma who underwent two surgeries were included in this study; the first surgery was at diagnosis, and the second surgery was after relapse. All patients signed a written informed consent form, and this study was approved by the Institutional Review Board (protocol # UCD 1412052).

Demographic and clinical information including age, sex, ethnicity, and MGMT methylation for the study subjects was obtained via medical record review. The patients received surgical resection, concurrent radiation therapy, and adjuvant chemotherapy following the standard-of-care treatment protocol [18]. Tumor recurrence was confirmed based on pathology tissue obtained via surgery at recurrence.

Blood samples were collected before the initial surgery at diagnosis (Pre-Surgery), two days after the initial surgery (Post-Surgery), prior to starting radiation therapy (Pre-Radiation), after completing radiation therapy (Post-Radiation), and then before the second surgery and after the second surgery.

### 2.2. Data Pre-Processing and Machine Learning Models

The samples before the first surgery and before the second surgery at recurrence were placed in one category, “Pre-Surgery”, and mapped as “0”. The samples after the first and second surgeries were placed in the “Post-Surgery” group and mapped as “1”. The samples before radiation were placed in the “Pre-Radiation” group and mapped as “2”, while the samples after radiation were placed in the “Post-Radiation” group and mapped as “3”. Three classification models were considered: multinomial logistic regression, random forest, and gradient boosting. The models were built using the Scikitlearn library and Python 3 [19].

The parameters for the multinomial logistic regression classifier were C = 1.0, maximum iterations = 100, penalty = 12, and solver = saga.

The gradient-boosting classifier algorithm was used with the following parameters: learning rate = 0.2, max depth = 5, and number of estimators = 40. Accuracy, precision, recall, F1-score, ROC-AUC, log loss, and confusion metrics were used to evaluated the performance of the classification models. Gini importance was used as measure of important features in the best-performing model.

### 2.3. Statistical Analysis

The statistical analysis was based on detected intensities of metabolites identified through retention time (RT)-m/z pair [20]. Raw data for each metabolite were normalized and auto-scaled to minimize batch-to-batch data variation. GraphPad Prism 9 (version 9.5.1, San Diego, CA, USA) was used for statistical analysis. A fold-change analysis of intensity differences using a cut-off value of 3.0 was performed among the compared groups: pre-surgery (PreS) vs. post-surgery (PostS) and pre-radiation (PreRad) vs. post-radiation (PostRad). An unpaired Student’s *t*-test was used to identify significantly altered metabolites between the compared groups. (****) denotes *p*-value < 0.0001, (***) *p* < 0.001), and (**) a *p*-value < 0.01.

## 3. Results

### 3.1. Patients and Samples

MGMT methylation is a good prognostic factor [21]. We determined that, out of the six patients, three had MGMT promoter methylation, and three were unmethylated. The cohort consisted of two females and four males; the median age at diagnosis was 56 years (Table 1); the median BMI at diagnosis was 28 kg/m^2^.

### 3.2. Metabolomic Profiling

We identified 157 unique metabolites using the retention index and mass spectral matching (Supplementary Table S1) [20]. We compared a total of 39 samples at four points in the patients’ treatment course as follows: the first time point was prior to the first or second surgery (pre-surgery), the second was after the first or second surgery (post-surgery), the third was prior to starting radiation therapy (pre-radiation), and the fourth time point was after finishing radiation (post-radiation).

**Figure 1:**
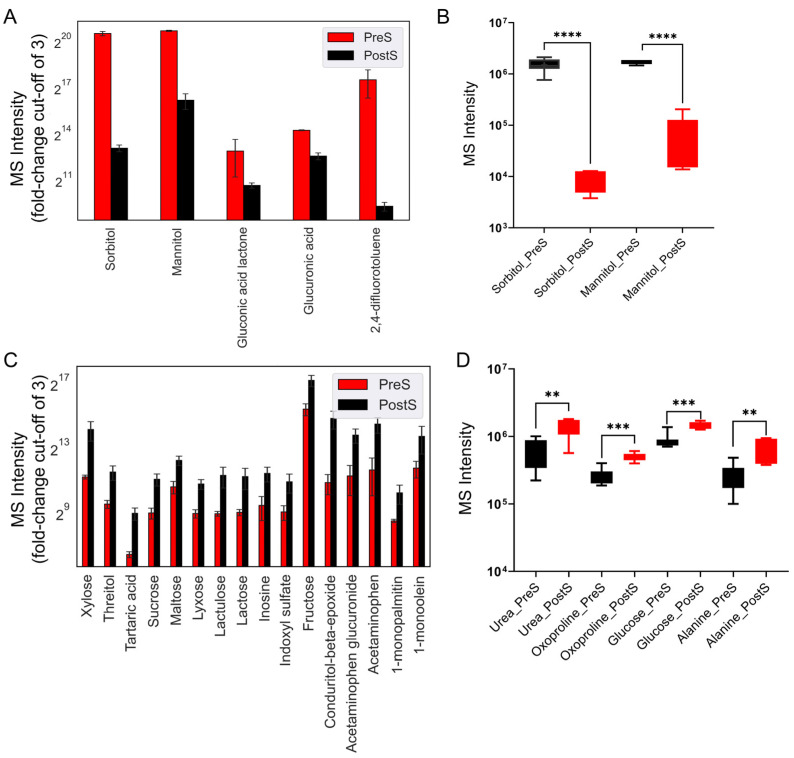
GC-TOF MS intensities of untargeted plasma metabolomics for pre-surgical and post-surgical samples. (**A**) Levels of decreased metabolites post surgery (PostS) compared to pre-surgery values (PreS) with a cut-off fold-change of 3. (**B**) Metabolites with a significant decrease post-surgery (*p* < 0.05). (**C**) Comparison of increased metabolite levels pre surgery vs. post surgery, with a cut-off fold-change of 3. (**D**) Metabolites with a significant increase post surgery (*p* < 0.05). Statistical significance was determined using an unpaired Student’s *t*-test, where (****) denotes a *p*-value < 0.0001, (***) *p* < 0.001, and (**) a *p*-value < 0.01.

To analyze the altered metabolites at different stages of treatment, we performed exploratory data analysis to capture the comparative profiles with an MS intensity fold-change cut-off of 3.0. Figure 1A illustrates the metabolites that decreased post surgery, while Figure 1C shows the metabolites which increased post surgery. The statistical analysis of the identified metabolites showed a significant decrease in sorbitol and mannitol (Figure 1B). In contrast, metabolites such as urea, oxoproline, glucose, and alanine significantly increased post surgery (Figure 1D).

**Figure 2:**
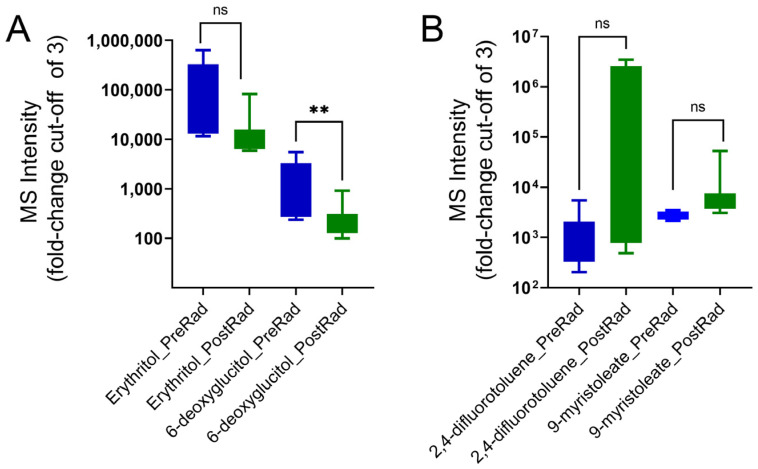
MS intensities of plasma metabolites for pre-radiation and post-radiation samples. (**A**) Levels of decreased metabolites post radiation (PostRad) compared to pre radiation (PreRad), with a cut-off fold-change of 3. Statistical significance was determined using an unpaired Student’s *t*-test. Metabolites with a significant decrease post radiation (** *p* < 0.01). (**B**) Increased metabolites post radiation with a cut-off fold-change of 3. “ns” Not significant.

Comparing pre-radiation to post-radiation samples, a decrease in 11 metabolites with a two-fold cut-off was found. Two metabolites, erythritol and 6-deoxyglucitol, showed a de-crease with a cut-off of three, with a significant reduction for 6-deoxyglucitol (Figure 2A). For the metabolites increasing post radiation, two metabolites, 2,4-difluorotoluene and 9-myristoleate, were identified with a cut-off of three but failed to achieve significance (*p* values of 0.053 and 0.405, respectively) (Figure 2B).

### 3.3. Correlation Analysis

**Figure 3:**
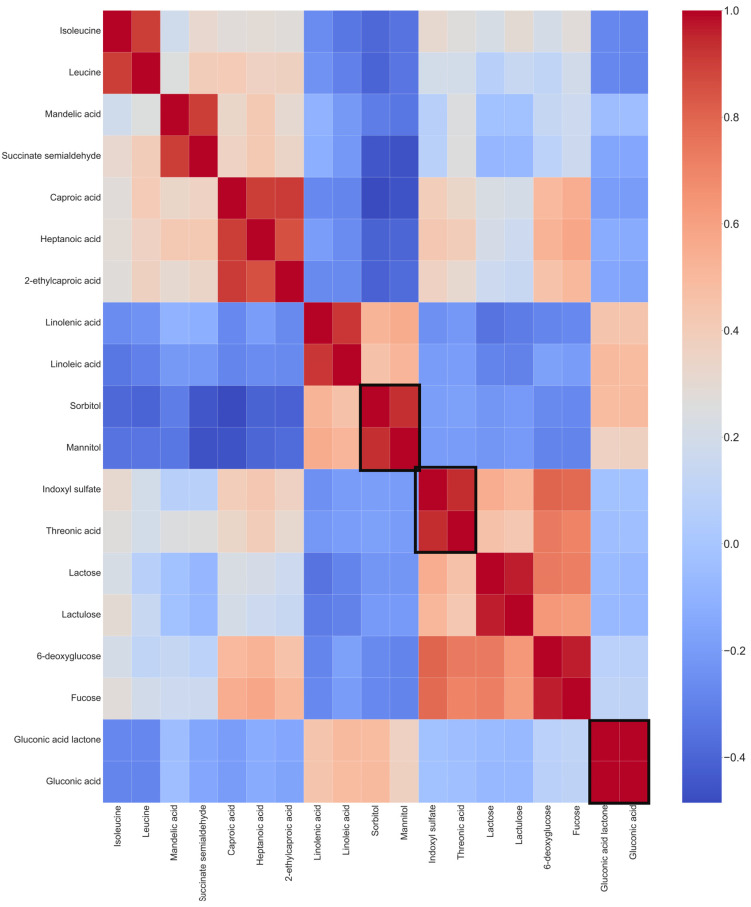
Heatmap of Pearson’s correlation coefficients for altered plasma metabolites with a cut-off of r > 0.90. Altered metabolites with high correlations are highlighted in black boxes. The correlation score can be tracked through the scale bar on the right side of the heatmap. Positive correlations are present between several metabolites: between sorbitol and mannitol, indoxyl sulfate and threonic acid, and gluconic acid and gluconic acid lactone.

We computed pairwise associations between 19 metabolites using Pearson’s correlation with a particular cut-off (positive pairs r > 0.90) to identify associations between metabolite changes from the altered metabolites. As shown in (Figure 3), correlations were detected between several metabolites. Notably, positive correlations for altered metabolites included sorbitol and mannitol, indoxyl sulfate and threonic acid, and gluconic acid and gluconic acid lactone (Figure 3).

### 3.4. Machine Learning Models for Evaluating Tumor Stage

To understand tumor prognosis in patients undergoing two surgeries using serum metabolites, we implemented three machine learning algorithms for classification: multinomial logistic regression (MLR) [22], gradient boosting (GB classifier), and random forest.

**Figure 4:**
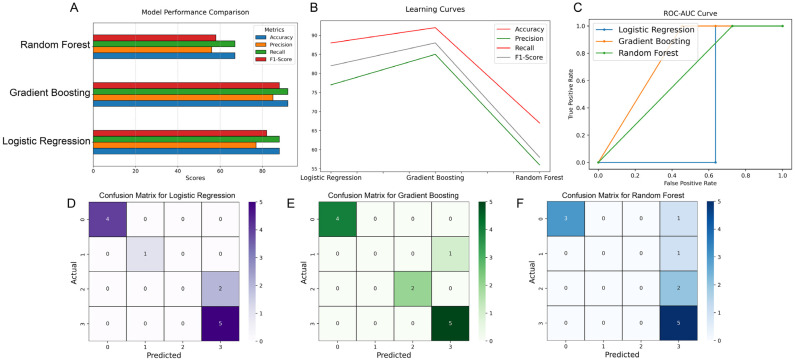
Metabolomics with machine learning models for the classification of clinical stages in patients with recurrent glioblastoma undergoing repeat surgery. (**A**) Comparing the performance of metabolomic-based machine learning algorithms based on accuracy, precision, recall, and F1-score. (**B**) The learning curve on test samples as a function of the training samples. (**C**) ROC-AUC curve to assess the performance of the three models. (**D**–**F**) Confusion matrix for each of the three models when tested on the test dataset consisting of 12 samples. The color scales (0–5) next to each confusion matrix represent classification accuracies. The actual/prediction labels are mapped as follows: “0” for pre surgery, “1” for post surgery, “2” for pre radiation, and “3” for post radiation.

For the three models, the dataset was split at a 70:30 train-to-test ratio. The performance of the classification models was tested using accuracy, precision, recall, F1-score, and the ROC-AUC curve. As shown in Figure 4A, the gradient-boosting and logistic regression models were superior to random forest. Furthermore, gradient boosting revealed a better performance than logistic regression in all performance scores (accuracy: 92% vs. 88%; precision: 85% vs. 77%; recall: 92% vs. 88%; F1-score: 88% vs. 82%; and ROC-AUC: 100% vs. 85%) (Figure 4A,C). To measure the predictive performance of the three models, we computed the learning curve on test samples as a function of training (Figure 4B). As our interest was to develop an algorithm that resulted in the correct classification of samples, we computed the log-loss to measure the prediction probability of both logistic regression and gradient boosting. As shown in Supplementary Figure S1, the gradient-boosting model exhibited a much lower log-loss of 0.096 compared to logistic regression (0.649), indicating a superior performance for the prediction of tumor conditions in patients with GB who have undergone repeat surgeries. For GB classification, the Gini importance of the top ten metabolite features was calculated. The most important features were mannose, isoleucine, ribonic acid, and 2,4-difluorotoluence, with a Gini importance greater than 0.1, followed by arachidonmic acid (0.039477) and threitol (0.036550), and, to a lower extent, 3-aminoisbutryric acid, 2-ethylcaproic acid, glycolic acid, and phenylacetic acid, with a Gini importance range between 0.024195 and 0.017581.

To study the ability of each of the three models to classify samples in the correct tumor stage, we used a 4 × 4 confusion matrix (Figure 4D–F). The multinomial logistic regression algorithm correctly classified 10 out of 12 samples, with 100% accuracy for classifying them into pre and post surgery.

The gradient-boosting classifier, on the other hand, classified 11/12 samples correctly. The gradient-boosting algorithm showed the most accurate classification of pre-radiation vs. post-radiation samples, where 7/7 samples were correctly classified by the GB model versus 5/7 by the logistic regression model. The random forest algorithm exhibited the lowest accuracy in classifying pre-surgery versus post-surgery samples.

## 4. Discussion

This study elucidates significant metabolomic changes associated with patients with recurrent IDH-wildtype glioblastoma undergoing surgical resection and radiation therapy. By analyzing plasma metabolites, we identified distinct metabolic alterations across different treatment stages, shedding light on the complex biochemical landscape of this aggressive cancer. Our findings underscore the potential of metabolomic profiling combined with machine learning to improve the understanding of tumor behavior. Our metabolomic analysis revealed significant alterations in various metabolites post surgery and post radiation therapy. Notably, we observed a decrease in the sorbitol and mannitol levels post surgery; such a change was noted after both surgeries in each patient, consistent with these metabolites’ roles in cellular osmotic regulation and oxidative stress response. Our findings support the results by Kucharzewska et al. demonstrating increased sorbitol levels due to the activation of the polyol pathway, in hypoxic glioma cells. The decrease in sorbitol levels post surgery suggests an important role for non-glycolytic metabolic pathways in hypoxic glioblastoma cells. The decrease in sorbitol levels post tumor resection may be associated with the decreased oxidation of sorbitol to fructose and the decreased production of NAD following tumor removal. This is in line with several studies that showed that the inhibition of the NAD metabolism reduces the survival of glioma cells and overcomes TMZ resistance in glioblastoma [23]. Elevated levels of urea, oxoproline, glucose, and alanine after surgery suggest increased protein catabolism and gluconeogenesis, reflecting the body’s metabolic adaptation to surgical stress and tumor burden.

Interestingly, mannitol has been associated with lower intracranial pressure [24]. It is possible to suggest that the decrease in mannitol post surgery is due to a reduced tumor burden in patients with glioblastoma after resection. Mannose, involved in glycosylation, and mannitol, a regulated metabolite derived from mannose, may contribute to osmotic balance and stress response in tumor cells. Isoleucine supports biosynthetic processes necessary for tumor growth, while ribonic acid is involved in nucleotide biosynthesis. These interconnected metabolites may suggest potential metabolic adaptations that may influence glioma progression. In addition, several metabolites involved in the pentose phosphate pathway, such as glucuronic acid lactone and gluconic acid, showed decreased levels post surgery.

An interesting study by Radenkovic et al. demonstrated an increase in mannitol in the blood and urine samples of patients with congenital disorders of glycosylation (CDGs) suggesting an association between abnormal glycosylation and polyol metabolism. Schwab et al. suggested that the polyol pathway is an important marker linking glucose metabolism to cancer aggressiveness [25].

The post-radiation analysis demonstrated a decrease in eleven metabolites with a fold-change cut-off of two, and a decrease in two metabolites with a cut-off of three. The statistical analysis showed a significant reduction in 6-deoxyglucitol post radiation, while erythritol failed to achieve significance. Our data agree with a previous study by Björkblom et al. [26], who observed that erythritol plays an important role in brain cancer development through the regulation of hydrogen peroxide. Interestingly, 6-deoxyglucitol, which can be reduced to erythritol, showed a significant decrease post radiation. On the other hand, our data demonstrated an increase in two metabolites post radiation—2,4-difluorotoluene and 9-myristoleate. The involvement of 2,4-difluorotoluene in tumor progression is not clear; however, our Gini importance suggest that it is an important feature of model prediction. The increase in 9-myristoleate can be associated with the altered fatty acid (FA) metabolism of glioblastomas [27].

The correlation analysis identified significant positive associations between metabolites such as sorbitol and mannitol, indoxyl sulfate and threonic acid, and gluconic acid and gluconic acid lactone. These correlations suggest coordinated metabolic responses, possibly reflecting shared regulatory mechanisms or pathways. Notably, the involvement of sugar alcohols and amino acids in these correlations points towards alterations in osmotic balance and the nitrogen metabolism, which are crucial for cancer cell survival and proliferation.

The pathway analysis indicated that these altered metabolites are involved in critical pathways such as the pentose phosphate pathway, glycolysis, and amino acid metabolism. The observed changes in these pathways align with known metabolic reprogramming in cancer, where cells shift their metabolic flux to support rapid growth and evade immune responses.

The selected untargeted metabolomics approach has significant advantages as it allows a comprehensive analysis of all metabolites compared to measuring well-defined groups of metabolites in targeted metabolomics [28]. The comprehensive detection of all metabolites in a sample generates complex data that can be analyzed using ML tools to help predict clinical outcomes. Our study leveraged ML algorithms to classify the metabolic profiles associated with different treatment stages.

Multinomial logistic regression is commonly used to predict an outcome in multiple categories from different dependent predictors. Random forest is an ensemble-learning method for classification, consisting of multiple decisions trees and less prone to overfitting. Gradient boosting is one of the most powerful ensemble algorithms for classification. This algorithm grows decision trees based on the prediction errors of the previous trees to improve the overall performance accuracy. The gradient-boosting classifier outperformed multinomial logistic regression and random forest in distinguishing pre- and post-surgical, as well as pre- and post-radiation, states. This finding underscores the robustness of gradient boosting in handling complex, non-linear relationships inherent in metabolomic data.

The superior performance of gradient boosting, evidenced by its higher accuracy, precision, and recall, highlights its potential for developing predictive models that can assist clinical decision making. By accurately classifying treatment stages, these models can provide insights into patient prognosis by predicting early recurrence and help tailor individualized treatment plans.

Despite the promising findings, our study has several limitations. The small sample size and single-center data collection restrict the generalizability of our results. The rarity of recurrent IDH-wildtype glioblastoma and the challenges of conducting longitudinal studies in this patient population contributed to the limited cohort size, which may increase susceptibility to overfitting in our models. Future studies should aim to include larger, multi-center cohorts to validate our findings and enhance model robustness. Furthermore, the absence of a control group, such as patients with benign tumors or those undergoing elective non-tumor surgeries, limits our ability to contextualize the observed metabolic changes against a broader clinical background; we plan to incorporate such controls in future studies. Additionally, perioperative medications, especially corticosteroids administered as standard care, may influence metabolomic profiles and potentially confound specific metabolite levels. While mannitol was not routinely administered, we acknowledge that corticosteroid use could impact certain metabolites and are exploring methods to control for these factors. Lastly, none of the patients in our study had documented metabolic disorders, such as poorly controlled diabetes, prior to their initial diagnosis which could further influence the baseline metabolite levels.

Further research should explore integrating additional clinical features such as BMI, race, age, and gender, which may influence metabolic responses and treatment outcomes. Moreover, while our models effectively classified treatment stages, their utility in predicting long-term treatment responses and tumor recurrence remains to be established.

## 5. Conclusions

In conclusion, our study demonstrates the utility of metabolomic profiling and machine learning in uncovering metabolic alterations in recurrent IDH-wildtype glioblastoma. The identified metabolites and associated pathways provide valuable insights into this tumor’s metabolic landscape and potential therapeutic targets. Our findings pave the way for future research to develop more personalized and effective treatment strategies for patients with glioblastoma.

## Figures and Tables

**Table 1:** Patient demographics and sample groups. “M”, male; “F”, female; “N”, MGMT promoter unmethylated; and “P”, MGMT promoter methylated. BMI in kg/m^2^. “X” represent sample collection.

Patient #	Gender	Age at Diagnosis	BMI at Diagnosis	Pathological Diagnosis	MGMT	Pre-Surgery	Post-Surgery	Pre-Radiation	Post-Radiation
1	M	60	40	Glioblastoma, IDH wildtype	N	XX	XX	X	XXXX
2	M	43	28	Glioblastoma, IDH wildtype	P	X	XX		XXXX
3	M	47	36	Glioblastoma, IDH wildtype	P	X	XX	X	XXXXX
4	M	56	26	Glioblastoma, IDH wildtype	P	X	X	X	XXX
5	F	58	27	Glioblastoma, IDH wildtype	N	X		X	XXX
6	F	60	20	Glioblastoma, IDH wildtype	N	X	X	X	

## Data Availability

All data can be made available upon request.

## Supplementary Materials

The following supporting information can be downloaded at https://www.mdpi.com/article/10.3390/cancers16223856/s1: Figure S1: Pie chart showing fractions of logarithmic loss or cross-entropy loss for gradient-boosting, logistic regression, and random forest classification algorithms; Table S1: Data sheet of all the metabolites detected across all the analyzed samples.
