# Supplementary material for: Metabolomic Profiling and Machine Learning Models for Tumor Classification in Patients with Recurrent IDH-Wild-Type Glioblastoma: A Prospective Study
Source: Cancers (Basel). 2024 Nov 17;16(22):3856. doi: 10.3390/cancers16223856 (PMC11593314; doi:10.3390/cancers16223856)
Supplement: Supplementary file 1 [file cancers-16-03856-s001.zip › cancers-3258870-supp Figure S1.pdf]

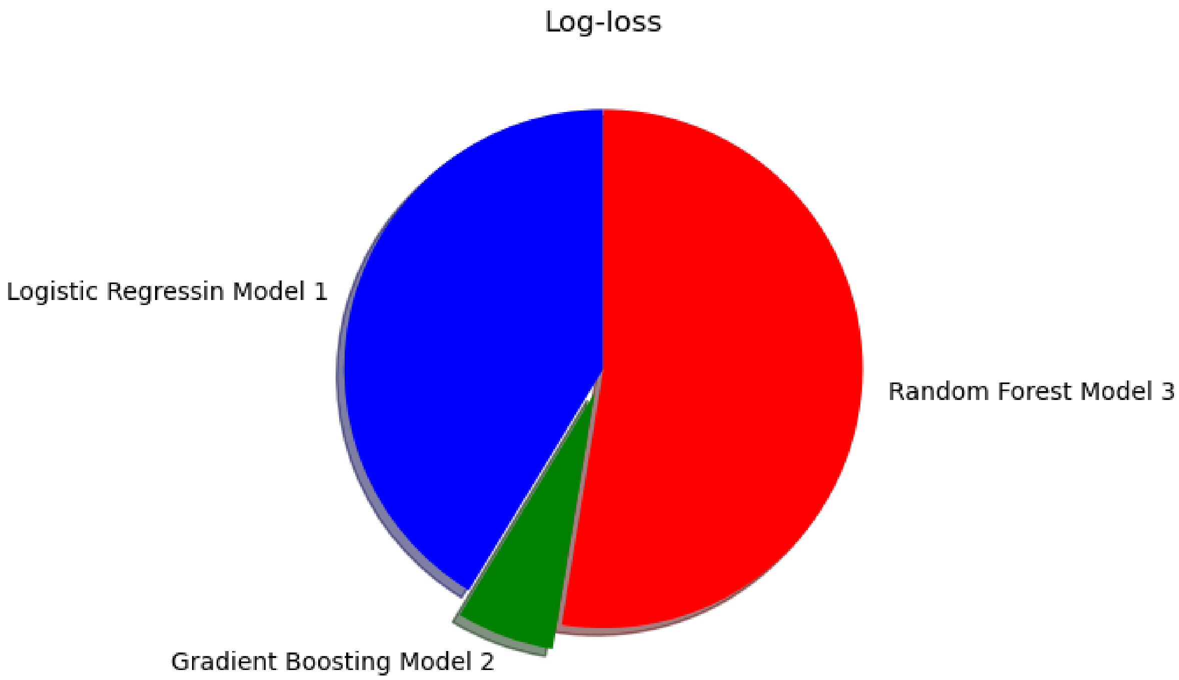

Figure S1. Pie chart showing fractions of logarithmic loss or cross-entropy loss for gradient boosting, logistic regression, and random forest classification algorithms.
